# Prevalence and predictors of self-reported hearing aid use and benefit in Norway: the HUNT study

**DOI:** 10.1186/s12889-024-17852-z

**Published:** 2024-02-15

**Authors:** Bo Engdahl, Lisa Aarhus

**Affiliations:** 1 Department of Physical Health and Ageing, Norwegian Institute of Public Health, Postbox 4404 Nydalen, N-0403 Oslo, Norway; 2 Department of Occupational Medicine and Epidemiology, National Institute of Occupational Health, Oslo, Norway; 3 Department of Internal Medicine, Diakonhjemmet Hospital, Oslo, Norway

**Keywords:** Hearing loss: tinnitus, Treatment, Ageing, Marital status, Socioeconomics

## Abstract

**Background:**

Knowledge on hearing aid use and benefit is important to ensure appropriate and effective treatment. We aimed to assess prevalence and predictors of hearing aid use and benefit in Norway, as well as possible birth cohort changes.

**Methods:**

We analyzed two large cross-sectional, population-based hearing surveys of 63,182 adults in 1996–1998 and 2017–2019 (the HUNT study). We used multivariable regression models to examine independent predictors of hearing aid use and benefit, including demography, hearing-related variables, known risk factors for hearing loss and birth cohort.

**Results:**

The nationally weighted hearing aid use in the adult population increased from 4.2% in 1997 to 5.8% in 2018. The use among individuals with disabling hearing loss (≥ 35 dB HL) increased from 46.3% to 64.4%. Most users reported some (47%) or great (48%) help from their hearing aids. In addition to the level of hearing loss and birth cohort, factors associated with hearing aid use included lower age, tinnitus, childhood-onset hearing loss, higher education, marriage, having children, being exposed to occupational noise or impulse noise, recurrent ear infections, and head injury. In addition to the level of hearing loss, factors related to hearing aid benefit included younger age, female gender, and higher income. Being bothered by tinnitus reduced the benefit.

**Conclusion:**

Our study shows an increase in self-reported hearing aid usage over time in Norway, with lower adoption rates and perceived benefits observed among the elderly. The results suggest that having a spouse and children positively influences the adoption of hearing aids. These findings emphasize the necessity of customized strategies to address demographic disparities and the need for innovative enhancements in hearing rehabilitation programs.

**Supplementary Information:**

The online version contains supplementary material available at 10.1186/s12889-024-17852-z.

## Introduction

Hearing loss is a common disability that affects a significant portion of the population, particularly older individuals. The process of hearing rehabilitation is crucial to manage the difficulties and consequences associated with hearing loss. Hearing rehabilitation focuses on enhancing communication and safety and to improve the overall quality of life [1], and it may provide cognitive benefits [2].

Hearing aids are commonly utilized in hearing rehabilitation to address hearing loss. The need for hearing aids is influenced by several factors beyond the type and severity of hearing loss. Considerations such as communication needs, lifestyle and activities, emotional and psychological well-being, cognitive function, support network, individual preferences, and financial considerations all play a role in determining the need for rehabilitation with hearing aid [3–5].

Moreover, the effectiveness of hearing aids depends on auditory factors, such as hearing loss type and severity, as well as non-auditory factors, including age, overall health, individual preferences and needs, and the availability of support. Especially older adults face unique challenges due to the cumulative impact of age-related conditions, such as cognitive decline or physical limitations, which can complicate the operation and maintenance of hearing aids. Hearing rehabilitation may also include the use of assistive hearing devices and aural rehabilitation such as counseling, auditory training and communication strategies although the evidence in support of aural rehabilitation for older adults with hearing loss has also been questioned [6].

Most population studies conclude that hearing aid adoption rates remain low and highlight a substantial unmet need for treatment [7–9]. However, quantifying the unmet need for treatment requires consideration of not only the prevalence of treatment, but also the needs and effectiveness of hearing aids. To our knowledge, no previous study has assessed predictors of both hearing aid use and benefits in the same cohort.

We analyzed two large cross-sectional, population based hearing surveys of Norwegian adults conducted 20 years apart. Our aim was to obtain data on the age-specific prevalence and potential birth cohort changes in hearing aid use. Additionally, we investigated factors associated with hearing aid use and self-reported benefits of use.

## Methods

### Participants

The Trøndelag Health (HUNT) Study is a large general health-screening study for the entire adult population of Nord-Trøndelag County, Norway. It consists of four surveys conducted between 1984 and 2019 [10]. Nord-Trøndelag is fairly representative of Norway except for the lack of large cities and immigrant populations [10]. We used data from two hearing surveys: HUNT2 Hearing (1996–1998) and HUNT4 Hearing (2017–2019).

HUNT2 Hearing included 17 of the 24 municipalities in the county. The participation rate was 63%, and a total of 51,529 persons attended. Valid pure-tone audiometry and data from a questionnaire that was distributed to all participants and returned at the site of the examination were available for 49,594 participants. HUNT4 Hearing was carried out in the six larger municipalities, representing approximately two-thirds of Nord-Trøndelag County. The participation rate was 43%, and a total of 28,388 persons attended. The hearing studies are described in detail elsewhere [11, 12]. After excluding persons with missing questionnaires or non-valid pure-tone audiometry, the final cross-sectional samples comprised 49,594 and 26,606 participants in HUNT2 respectively HUNT4. The number of subjects participating in both HUNT2 and HUNT4 Hearing were 12,115.

### Measurement

In addition to the questionnaires, both hearing studies included the same otoscopy and audiometric procedure. Pure-tone air-conduction hearing threshold levels were determined following the procedure described in ISO 8253–1, with fixed frequencies at eight test frequencies between 0.25–8 kHz using an automatic procedure with the ascending method. Hearing thresholds were defined relative to the hearing threshold levels of a population of otologically normal subjects aged 19–23 years. This is to compensate for possible systematic differences in calibration between audiometry in HUNT2 and HUNT4 and the departures from ISO 389 as previously recorded for the TDH-39P earphones [12].

#### Outcome measures

We analyzed two outcome variables, use of hearing aids and hearing aid benefits. The use of hearing aids was measured by the question: “Do you use a hearing aid?” (yes/no) and was obtained next to a filter-question about self-reported hearing loss: “Do you have a hearing loss that you are aware of?” (HUNT2), “Do you believe you have impaired hearing?” (HUNT4). Participants with self-reported hearing loss who missed data on the following question on use of hearing aids, were treated as no use of hearing aid if their measured hearing was normal (*n* = 1,734 in HUNT2 and 340 in HUNT4). Otherwise, participants with self-reported hearing loss and missing hearing aid data were excluded (*n* = 1,044 in HUNT2 and 366 in HUNT4).

The self-reported benefit of hearing aid was obtained only in HUNT4. Only participants first reporting use of hearing aid were included. It was measured with the single item, “How much help do you have from your hearing aid?”, in four categories: no help, some help, great help and removes all problems. Out of 1,751 users only 19 had missing information on self-reported benefit.

#### Explanatory variables

We investigated several explanatory variables: demography (age, education, and income), hearing-related factors (hearing threshold, tinnitus), risk factors for hearing loss (occupational and impulse noise, head injury, recurrent ear infection) and birth cohort (only for the outcome hearing aid use).

Pure-tone average hearing threshold (PTA4) was determined as the average hearing thresholds of 0·5, 1, 2 and 4 kHz in the better-hearing ear in dB HL. The severity of hearing loss was defined using the criteria for classification by WHO (WHO Stevens et al. 2013; Wilson et al. 2017) in 15 dB intervals from good hearing (< 20 dB) in the better ear, to total impairment (≥ 95 dB) in the better ear with disabling hearing loss defined as PTA4 ≥ 35 dB HL. Self-reported hearing loss was measured by the questions: “Do you have a hearing loss that you are aware of?” (HUNT2), “Do you believe you have impaired hearing?” (HUNT4).

Information on childhood-onset hearing loss (hearing loss diagnosed by an ear-nose and throat specialist as sensorineural or related to chronic suppurative otitis media, recurrent ear infections or otosclerosis) with PTA4 =  > 25 dB HL was obtained in a subsample born between 1940 and 1980 from the School Hearing Investigation in Nord-Trøndelag (SHINT), an audiometric screening of all schoolchildren attending regular schools in the County of Nord-Trøndelag from 1954 to 1986 [13].

Tinnitus was defined as tinnitus that is experienced daily or almost always, with periods lasting more than 5 min (HUNT4) or 10 min (HUNT2) and experienced as bothersome.

We obtained the following information from national registers: education (primary school, secondary school, university < 4 years, university >  = 4 years), occupation (white-blue collar), pensionable income standardized on age and cohort, marital status and having children. White-collar/blue-collar occupation was based on the Norwegian version of the International Standard Classification of Occupations, ISCO88, with one-digit level codes 1–5 categorized as white-collar and codes 0, and 6–9 as blue-collar workers.

From similar questions in HUNT2 and HUNT4, we obtained estimates of risk factors for hearing loss: occupational noise (regularly been exposed to loud noise at your present or previous work [no/less than 5 h/week, >  = 5 h/week]), impulse noise (more often than most people, been exposed to impulse noise (explosions, shooting etc.) [no, maybe, yes]), recurrent ear infections (no, maybe, yes), and hospitalization for head injuries (no, maybe, yes). The maybe category was coded as no exposure. We treated missing values on any of these risk factors as no exposure, which accounted for < 5% in each variable.

### Statistical analysis

Statistical tests were calculated in Stata version 17.0 with 95% confidence intervals. The alpha level was set at 0.05 for all analyses.

#### Prevalence of hearing aid use

We presented the prevalence of use of hearing aids as a function of hearing loss in the two cohorts. To provide nationally weighted population estimates for adults over 19 years of age in Norway we accounted for the age and sex distribution of the Norwegian population in 1997 and 2018, by applying weights obtained from Statistics Norway [14]. To compare the prevalence of hearing aid use and prevalence of hearing loss (≥ 35 dB HL) across age and cohort, we applied logistic models including sex, age, cohort and interaction between cohort and age. Probabilities along with their corresponding 95% confidence intervals were predicted using the margins command in Stata.

#### Predictors of hearing aid use and benefit of hearing aids

We used logistic regression to examine predictors of hearing aid use (yes/no) in the pooled cross-sectional sample. All explanatory variables were included in the model. Hearing threshold, age, education, and income were treated as continuous variables. To reveal cohort-specific associations we investigated two-way interactions between cohort and hearing (hearing threshold and tinnitus), age and sex. We also tested for two-way interactions between hearing threshold, age, and sex to explore if the association between hearing loss and use or benefit varied with age and sex.

We used ordinal regression to assess predictors of hearing aid benefit (four ordered categories) in the HUNT4 sample. We performed the same analyses as for hearing aid use, except the analyses including birth cohort.

To estimate the frequency of hearing thresholds that best predicted the use and benefit of hearing aids, we applied the same multivariable regression models replacing the PTA4 threshold with each 8 frequencies from 250 to 8000 Hz as independent variables. Finally, we investigated the effect of permanent childhood hearing loss in a subsample consisting of individuals born between 1940 and 1980.

To account for the dependence in the pooled data resulting from subjects participating in both surveys, we employed cluster-robust standard errors, utilizing the sandwich estimator.

## Results

### Characteristics of the sample

The final sample included 63,182 participants with 75,190 observations divided into 48,676 in HUNT2 and 26,514 in HUNT4. Age ranged from 20 to 99 years (mean = 51.2 (50.0 in HUNT2/ 53.5 in HUNT4)) with 46% men (47%/44%). The prevalence of disabling hearing loss (≥ 35 dB HL) was 6.3% (6.6%/5.6%) and of self-reported hearing loss 32% (26%/41%). The relation between self-reported hearing loss (yes/no) and PTA4 hearing threshold (dB HL) measured as point-biserial correlation was 0.43 (0.44/0.44).

#### Hearing aid use

Table 1 displays the characteristics of the sample by hearing aid use and benefit. Users of hearing aids were older and had higher hearing thresholds. There were more users of hearing aids in men, married, those with children, with low education, poor hearing, tinnitus, being white collar worker, exposed to occupational noise, impulse noise, recurrent ear infections, and head injuries. The use of hearing aids increased from 4.2% (women 3.1%, men 5.4%) in the 1996–1998 cohort to 6.6% (women 5.0%, men 8.6%) in the 2017–2019 cohort.

**Table 1:** Description of participants as a function of use of hearing aids (total observations = 75,190 participants = 63,182)

	HUNT2 Hearing (1996–1998)				HUNT4 (2017–2019)			
	Hearing aid use				Hearing aid use			
	No		Yes		No		Yes	
N	46,646 (95.8%)		2,030 (4.2%)		24,763 (93,4%)		1,751 (6.6%)	
PTA4^a^ (dB HL), mean SD	9.6	11.4	43.6	17.0	8.5	10.3	36.1	14.9
Self-reported hearing loss^b^	9,222	22.7%	1,777	96.1%	9,193	37.4%	1,730	99.5%
Tinnitus	2,401	5.1%	382	18.8%	1,358	5.5%	363	20.7%
Age (years), mean SD	49.1	16.3	69.9	13.8	52.3	16.5	70.5	11.8
Male	21,526	46.1%	1,224	60.3%	10,570	42.7%	997	56.9%
Married	28,565	61.2%	1,289	63.5%	12,866	52.0%	1,155	66.0%
Having children	42,110	90.3%	1,754	86.4%	20,477	82.7%	1,640	93.7%
Education								
Primary school	14,135	30.3%	1,029	50.7%	3,354	13.5%	333	19.0%
Secondary	24,084	51.6%	835	41.1%	12,002	48.5%	924	52.8%
University < 4 years	7,146	15.3%	139	6.8%	7,559	30.5%	390	22.3%
University > = 4 years	1,281	2.7%	27	1.3%	1,848	7.5%	104	5.9%
Income (z-score), mean SD	0.00	1.00	-0.04	0.97	0.00	1.01	-.03	1.87
Blue collar	8,219	17.6%	94	4.6%	3,100	12.5%	102	5.8%
Occupational noise	10,883	23.3%	736	36.3%	4,396	17.8%	497	28.4%
Impulse noise	6,779	14.5%	523	25.8%	4,281	17.3%	550	31.4%
Head injury	3,173	6.8%	197	9.7%	1,877	7.6%	143	8.2%
Recurrent ear infections	11,491	24.6%	646	31.8%	4,110	16.6%	394	22.5%

^a^*PTA4* Pure-tone average hearing threshold determined as the average hearing thresholds of 0·5, 1, 2 and 4 kHz in the better-hearing ear in dB HL

^b^Self-reported hearing loss was measured by the questions: “Do you have a hearing loss that you are aware of?” (HUNT2), “Do you believe you have impaired hearing?” (HUNT4)

All percentages are columns-wise except the one in parentheses that are row-wise

**Fig. 1:**
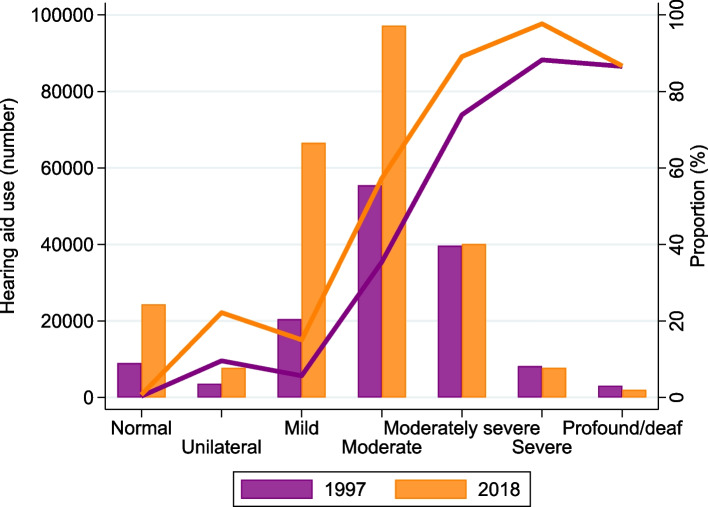
Estimated use of hearing aids in Norway after weighting on age- and sex for different degrees of hearing loss (WHO) based on self-reported use in HUNT2 and HUNT4 and population figures for Norway in 1997 and 2018. Shown are absolute numbers (bars) and proportions (lines). Number of adult inhabitants in Norway in 1997: 3.2 million and in 2028: 4.0 million

The estimated use in Norway after weighting on age- and sex increased from 4.2% (women 3.7%, men 4.7%) in 1997 to 5.8% (women 5.0%, men 6.6%) in 2018. Based on hearing aid use reported in HUNT4 and HUNT2, we can assume that approx. 230,000 people used hearing aids in Norway in 2018 and 130,000 in 1997. Figure 1 shows that there is a large increase in the number of hearing aid users among those with mild and moderate hearing loss.

#### Hearing aid benefit

**Fig. 2:**
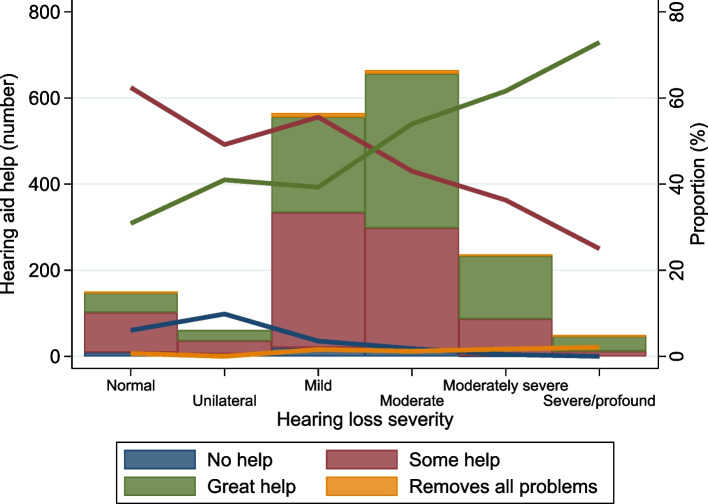
The distribution of different levels of hearing aid benefit for different degrees of hearing loss (WHO). Shown are absolute numbers (bars) and proportions (lines). Sample of hearing aid users in HUNT4 (*n* = 1,732)

The characteristics of the HUNT4 sample by hearing aid benefit (*N* = 1,732) are shown in Table 2. Most users of hearing aids reported that they had some (47%) or great help (48%) from their hearing aid. Only 24 users (1.4%) reported that the hearing aid removed all problems. Figure 2 shows that the self-reported benefit increases strongly by the level of hearing loss severity.

**Table 2:** Description of participants as a function of benefit of hearing aids (*N* = 1,732)

	Users of hearing aids in HUNT4 (2017–2019)							
	How much help do you have from your hearing aid?							
	No help		Some help		Great help		Remove all problems	
N	48 (2.8%)		822 (47.5%)		838 (48.4%)		24 (1.4%)	
PTA4^a^ (dB HL), mean SD	26.9	11.5	33.5	14.0	39.4	15.0	40.7	20.4
Tinnitus	8	16.7%	219	26.6%	167	19.9%	5	20.8%
Age (years), mean SD	64.9	16.0	70.6	11.6	71.0	11.6	67.1	12.8
Male	26	54.2%	512	62.3%	442	52.7%	10	41.7%
Married	24	50.0%	551	67.0%	554	66.1%	16	66.7%
Having children	44	91.7%	771	93.8%	783	93.4%	23	95.8%
Education								
Primary school	11	22.9%	156	19.0%	158	18.9%	3	12.5%
Secondary	25	52.1%	420	51.1%	457	54.5%	12	50.0%
University < 4 years	11	22.9%	192	23.4%	177	21.1%	7	29.2%
University > = 4 years	1	2.1%	54	6.6%	46	5.5%	2	8.3%
Income (z-score), mean SD	0.00	0.59	0.02	1.10	-0.02	0.92	0.03	1.08
Blue collar	5	10.4%	47	5.7%	46	5.5%	3	12.5%
Occupational noise	18	37.5%	244	29.7%	219	26.1%	8	33.3%
Impulse noise	15	31.3%	277	33.7%	246	29.4%	7	29.2%
Head injury	2	4.2%	65	7.9%	72	8.6%	1	4.2%
Recurrent ear infections	15	31.3%	181	22.0%	185	22.1%	7	29.2%

^a^ *PTA4* Pure-tone average hearing threshold determined as the average hearing thresholds of 0·5, 1, 2 and 4 kHz in the better-hearing ear in dB HL

All percentages are columns-wise except the one in parentheses that are row-wise

### Multivariate regression analyses

#### Estimated prevalences of hearing aid use and disabling hearing loss (Fig. 3)

**Fig. 3:**
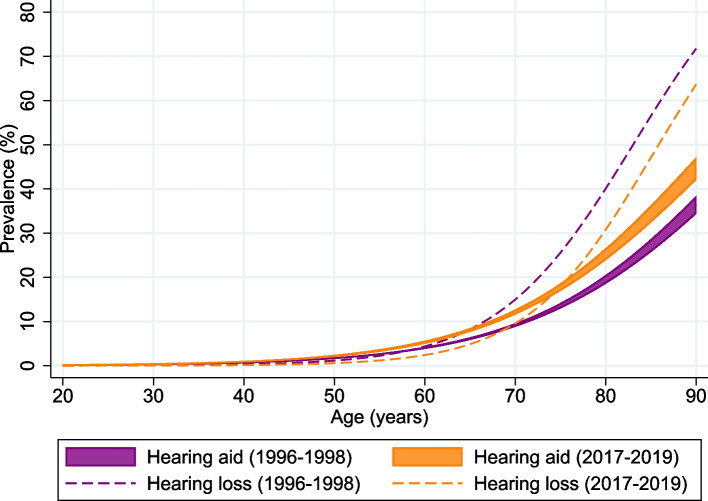
Predicted prevalence values of self-reported use of hearing aids and disabling hearing loss (PTA4 ≥ 35 dB HL) in HUNT2 and HUNT4. Prevalence values are probabilities predicted with 95% confidence intervals using the margins command in Stata from a logistic regression model including sex, age cohort and interaction between age and cohort

We used logistic regression analysis and the margins command to compare hearing aid use and prevalence of disabling hearing loss (≥ 35 dB HL) as a function of age. The proportion with disabling hearing loss was considerably larger than the proportion who used hearing aids in the 1996–1998 cohort. In the 2017- 2019 cohort there were more users of hearing aids than subjects with disabling hearing loss for participants younger than 75 years. Among the elderly, the proportion of hearing aid users was still less than the proportion with disabling hearing loss. The overall use among participants with disabling hearing loss increased from 46.3% in the 1996–1989 cohort to 64.4% in the 2017–2019 cohort. Values weighted by the Norwegian population in 1997 and 2018 were 46.8% and 60.4% respectively. The corresponding use among participants with self-reported hearing loss was 16.2% and 15.8% in the two cohorts and 16.7% and 14.6% respectively weighted by the Norwegian population.

#### Association between hearing aid use and various predictors (Fig. 4)

**Fig. 4:**
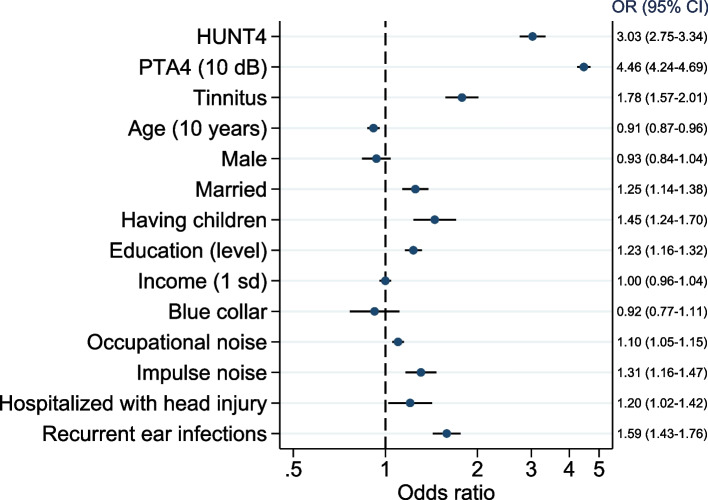
Associations between various explanatory variables and hearing aid use presented as adjusted odds ratio (OR) with 95% confidence intervals for all independent variables in the logistic regression model. For continuous variables the OR are for one-unit change with the unit shown in parentheses. Pooled sample from HUNT2 and HUNT4 (observations = 75,190, participants = 63,182)

Multiple logistic regression found lower age, higher hearing threshold and the HUNT4 cohort to be strongly associated with the use of hearing aids. Additionally, tinnitus, higher education, having children, being married, and being exposed to occupational noise, impulse noise, recurrent ear infections, and head injury all were independently associated with using hearing aid. Adding all hearing thresholds at single specific frequencies as independent variables showed that 1000 Hz, 2000 Hz, 4000 Hz and 500 Hz were the best predictors of the use of hearing aid (S-Fig. 1).

There was a significant interaction between cohort and hearing threshold (OR = 1.20 95% CI 1.10–1.30) with a 20% larger increase in the odds of using hearing aid per 10 dB increase in hearing threshold in the HUNT4 cohort compared with in HUNT2. There was also a significant interaction between cohort and tinnitus (OR = 1.42, 95% CI 1.13–1.78), with 42% higher odds of using hearing aids associated to tinnitus in HUNT4 cohort than in HUNT2. None of the other tested two-way interactions were significant.

Analyses of the subsample of those born between 1940 and 1980 showed that being diagnosed with childhood hearing loss increased the odds of using hearing aids independent on current hearing loss and other covariates (OR = 1.61, 95% CI 1.28–2.02).

#### Association between hearing aid benefit and various predictors (Fig. 5)

**Fig. 5:**
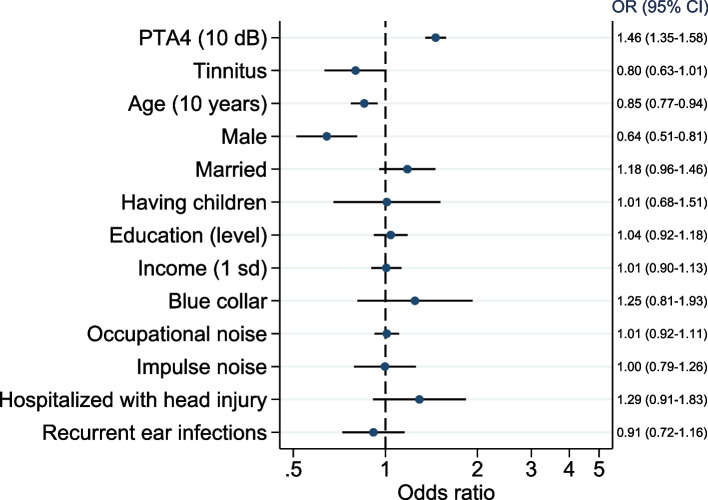
Associations between various explanatory variables and hearing aid benefit presented as adjusted odds ratio (OR) with 95% confidence intervals for all independent variables in the ordinal logistic regression model. For continuous variables the OR are for one-unit change with the unit shown in parentheses. Sample of hearing aid users in HUNT4 (participants = 1,732)

**Fig. 6:**
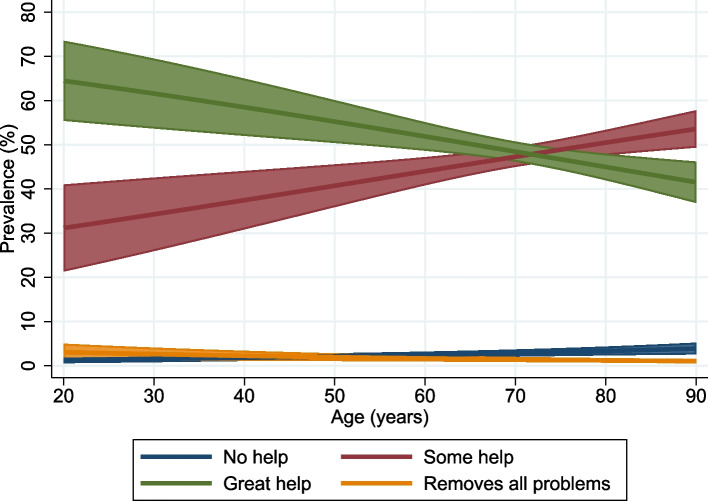
Predicted proportions of different levels of hearing aid benefit as a function of age. Proportions are predicted with 95% confidence intervals using the margins command in Stata from an ordinal logistic model including all independent variables. Sample of hearing aid users in HUNT4 (participants = 1,732)

Hearing aid benefit was associated with lower age, higher hearing threshold, being female, and having higher income. It was a negative association with being bothered by tinnitus. The association with age is illustrated in Fig. 6.

None of the tested two-way interactions were significant. The single frequency best predicting hearing aid benefit was 1000 Hz (S-Fig. 2). Analyses of the subsample of those born between 1940 and 1980 (*n*  = 1,247) showed that being diagnosed with childhood hearing loss only weakly increased the odds of benefitting from hearing aids independent on current hearing loss and other covariates (OR = 1.36, 95% CI 0.92–1.99).

## Discussion

### Principal findings

Our study shows an increase in the use of hearing aids among the more recent cohort (2017–2019) compared to the earlier cohort (1996–1998). Despite this increase, the proportion of individuals using hearing aids remained lower than the proportion of those with disabling hearing loss, particularly among the elderly.

Factors associated with use of hearing aids included hearing-related factors (severity of hearing loss, childhood-onset hearing loss, and tinnitus), sociodemographic factors (lower age, higher education, being married, and having children), and risk factors for hearing loss (exposure to occupational noise, impulse noise, recurrent ear infections, and head injuries).

Most users reported experiencing some or great help from their hearing aids. The extent of benefit was associated with the severity of hearing loss, being younger, being a woman, having higher income, and it was negatively associated with being bothered with tinnitus.

### Comparison to other studies

#### Prevalence of use of hearing aids and coverage rate

Our study showed an increase in hearing aid use during the last two decades, which align with previous studies. This increase is consistent with the rise in the number of people receiving hearing aids in somatic hospitals or from specialists in private practice, as documented in the Norwegian Patient Register [15]. Furthermore, there has been an increase in the number of hearing aids covered by the Norwegian Labour and Welfare Organization [15]. The respondents of the Eurotrak surveys of Norway reported an increase in use from 2012–2019 [16]. Similar increases in hearing aid adoption have been observed over the las two decades in the United States, France, and Germany, as reported by the MarkeTrak and Eurotrak surveys [17]. The increase was mainly driven by an increase in the number of hearing aid users among those with mild and moderate hearing loss.

Our study estimated that approximately 60% of participants with disabling hearing loss (≥ 35 dB HL) in the most recent cohort used hearing aids. This estimate is in line with a recent study that reported a 57% hearing aid coverage rate based on historical data on hearing aid sales in high-income countries among individuals with disabling hearing loss (> 40 dB), which constituted 4.6% of the population [18]. However, it is higher than the adoption rates of 33% among US adults with disabling hearing loss (≥ 35 dB) or reporting at least moderate hearing troubles [19].

It is important to note that studies including milder hearing losses tend to show lower coverage rates. For example, a previous analysis of the HUNT2 hearing study indicated that only 14% of adults above 65 years with bilateral hearing loss > 25 dB (62% of the sample) used hearing aids [3]. Another study by Popelka et al. [7] reported that 15% of subjects with better ear hearing loss ≥ 25 dB (which represented approximately 46% of the population [20]) used hearing aids.

Furthermore, the use of hearing aids among individuals with self-reported hearing loss tends to show lower coverage rates, which also depend on the severity of hearing loss or the prevalence in the sample. In our study, we found relatively low use (16% and 16% respectively) among participants with self-reported hearing loss, which was reported by 26% and 41% of the population in the respective cohorts. Eurotrak reported hearing aid use rates of 33–37% in Germany, France, and the UK among subjects with self-reported hearing loss affecting approximately 11% of the populations [21]. Prevalence estimates from American adults with self-reported hearing loss range from 27% in adults [22], 37% in adults aged 55 or older [4], to 45% in adults aged 65 or older [23].

#### Predictors of hearing aid use

Our study highlights the role of various factors in determining the use for hearing aid treatment beyond the severity of hearing loss. When considering the severity of hearing loss, the use of hearing aids decreased with age, which is a commonly observed trend. This decline may be attributed to several age-related factors, such as reduced benefit of hearing aids, different communication needs, increased challenges associated with hearing aid use, and potentially poorer identification of hearing loss among the elderly.

The impact of education on hearing aid use is consistent with findings from previous studies [3, 4, 7, 8, 23]. While income has been associated with use in other studies [4, 19, 23], we did not find it to be an independent factor in our study. This discrepancy may be explained by the liberal reimbursement rules in Norway, where hearing aids are provided free of charge through national health insurance. This may also be the reason for that having a blue collar work did not seem to be a barrier for assessing treatment with hearing aid. Surprisingly, we did not find evidence of lower usage among men, despite the fact that men typically exhibit a lower rate of help-seeking behaviour in general [24], The finding that marital status, particularly having a spouse, increases the use of hearing aids aligns with previous research, especially among men [3]. The observation that having children also increases the use of hearing aids is consistent with the notion that support from significant others plays a crucial role in seeking help for audiological problems.

The association between hearing aid use and tinnitus is expected, as hearing aids are commonly used for tinnitus treatment. Also, tinnitus was a more important factor for using hearing aids in the later cohort. The independent contributions of risk factors such as noise exposure, recurrent ear infections, and head injuries may relate to additional problems that increase the need for treatment. They may also increase the likelihood of being diagnosed with hearing loss. For instance, mandatory audiometric screening for workers in noise-related jobs increases the probability of treatment for individuals exposed to occupational noise. Presbycusis, the common age-related hearing loss unrelated to any known risk factors, typically exhibits a gradual decline, which can result in a longer time before it is identified.

#### Predictors of hearing aid benefits

The severity of hearing loss was the factor strongest associated with the benefit of hearing aid treatment. Previous studies have yielded inconsistent findings and reported weak associations with the degree of hearing loss [25]. It has been suggested that self-reported hearing loss may be a better predictor of hearing aid outcomes than measured hearing loss [26, 27]. Lower benefit of treatment among those with mild hearing loss may also be reflected in lower use.

Our study indicated that the benefit of hearing aid treatment extended beyond the severity of hearing loss. Older participants reported less help from their hearing aid than younger subjects at the same level of hearing loss. Although previous studies did not find sufficient evidence for age to be related to benefit [26, 28], benefits among older adults may be reduced due to challenges related to cognitive decline [29] or physical limitations. Women exhibited greater benefit, and there was an increased benefit among individuals with higher income. This conflicts with previous studies that did not find sufficient evidence for gender [26, 30, 31] or income [26] to be related to the benefit of hearing aid. Participants bothered by tinnitus reported less benefit of their hearing aids at a given severity of hearing loss, perhaps reflecting an extra burden or condition not as easily treated by hearing aids. This is not to say that hearing aid is not an effective treatment of tinnitus [32].

### Strength and limitations

Our study benefits from standardized audiometric measurements and questionnaire data obtained from a large population-based sample that is representative of an entire county of Nord-Trøndelag [10]. We believe Nord-Trøndelag is representative of Norway when it comes to hearing aid treatment as the county is at the national average in terms of the number of hearing aids dispensed per inhabitants adjusted for age and sex [33].

Like other large observational studies, there are some limitations to consider. The use and benefit of hearing aids were estimated using single-item questions. Although these questions had good face validity, they were not validated against any standardized instrument. Additionally, we did not have specific information related to the hearing aid fitting or delivery process, the type and quality of devices used, or the utilization of other personal sound amplification products and cochlear implants, which may have influenced our findings. A more detailed knowledge on the number of hours of usage and whether the fitting was uni/bilateral etc. would be needed to fully understand the associations of the various factors under investigation. We also acknowledge that self-reported benefits may not cover all possible benefits such as possible reduced social isolation, depression, cognitive decline, and other negative health outcome.

## Conclusions

In summary, our study reveals an increase in hearing aid usage over time, aligning with global trends. While hearing loss severity remains a critical factor in hearing aid use and benefit, several other factors also play crucial roles. Notably, we observed lower hearing aid use and benefit among elderly participants, potentially related to physical limitations and cognitive decline. Furthermore, our study emphasizes the significance of having a spouse and children, as we found that those without these significant others were less likely to use hearing aids. In addition, individuals with known risk factors of hearing loss showed higher adoption rates, emphasizing the importance of early detection and treatment.

These findings underscore the need for tailored approaches to address the diverse challenges faced by different demographic groups. Addressing the unmet need for hearing rehabilitation among the elderly may require innovative solutions, such as elder-friendly devices or specialized rehabilitation programs, to bridge the gap and enhance the quality of life for this population. To increase the adoption rate among individuals without a significant other, targeted interventions and support systems may be developed.

By considering these insights, healthcare professionals, policymakers, and researchers can develop more effective strategies to meet the diverse needs of individuals with hearing loss and improve hearing rehabilitation programs accordingly.

### Supplementary Information

**Additional file 1: S-Figure 1.** Hearing aid use estimated by a multivariable logistic regression model with hearing threshold at the frequencies 250-8 kHz as independent variables. HUNT2 and HUNT4. (Observations = 75,190 Participants = 63,182). Analyses adjusted for cohort, sex, and age. Odds ratio with 95% confidence intervals. **S-Figure 2.** Hearing aid benefit estimated by a multivariable ordinal logistic regression model with hearing threshold at the frequencies 250-8 kHz as independent variables. (N = 1,732).). Analyses adjusted for cohort, sex, and age. Odds ratio with 95% confidence intervals.

## Data Availability

The datasets generated and/or analyzed during the current study are not publicly available due to Norwegian legal restrictions and the current ethical approval for the study, but descriptive data in table form are available from the corresponding author on reasonable request.

## Abbreviations

HUNT: The Trøndelag Health Study
PTA: Pure-tone average hearing threshold
OR: Odds ratio
