# Supplementary material for: Prevalence and predictors of self-reported hearing aid use and benefit in Norway: the HUNT study
Source: BMC Public Health. 2024 Feb 15;24:474. doi: 10.1186/s12889-024-17852-z (PMC10867996; doi:10.1186/s12889-024-17852-z)
Supplement: Supplementary file 1 — Additional file 1: S-Figure 1. Hearing aid use estimated by a multivariable logistic regression model with hearing threshold at the frequencies 250-8 kHz as independent variables. HUNT2 and HUNT4. (Observations = 75,190 Participants = 63,182). Analyses adjusted for cohort, sex, and age. Odds ratio with 95% confidence intervals. S-Figure 2. Hearing aid benefit estimated by a multivariable ordinal logistic regression model with hearing threshold at the frequencies 250-8 kHz as independent variables. (N = 1,732).). Analyses adjusted for cohort, sex, and age. Odds ratio with 95% confidence intervals. [file 12889_2024_17852_MOESM1_ESM.pdf]

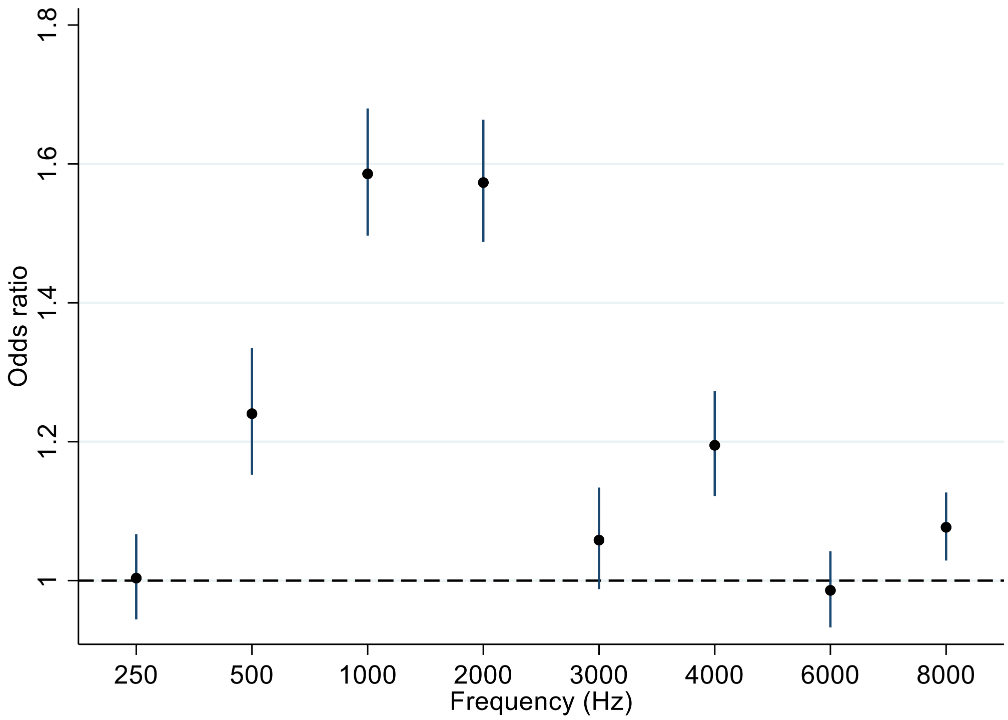

S-Figure 1: Hearing aid use estimated by a multivariable logistic regression model with hearing threshold at the frequencies 250-8 kHz as independent variables. HUNT2 and HUNT4. (Observations = 75,190 Participants = 63,182). Analyses adjusted for cohort, sex, and age. Odds ratio with 95% confidence intervals.

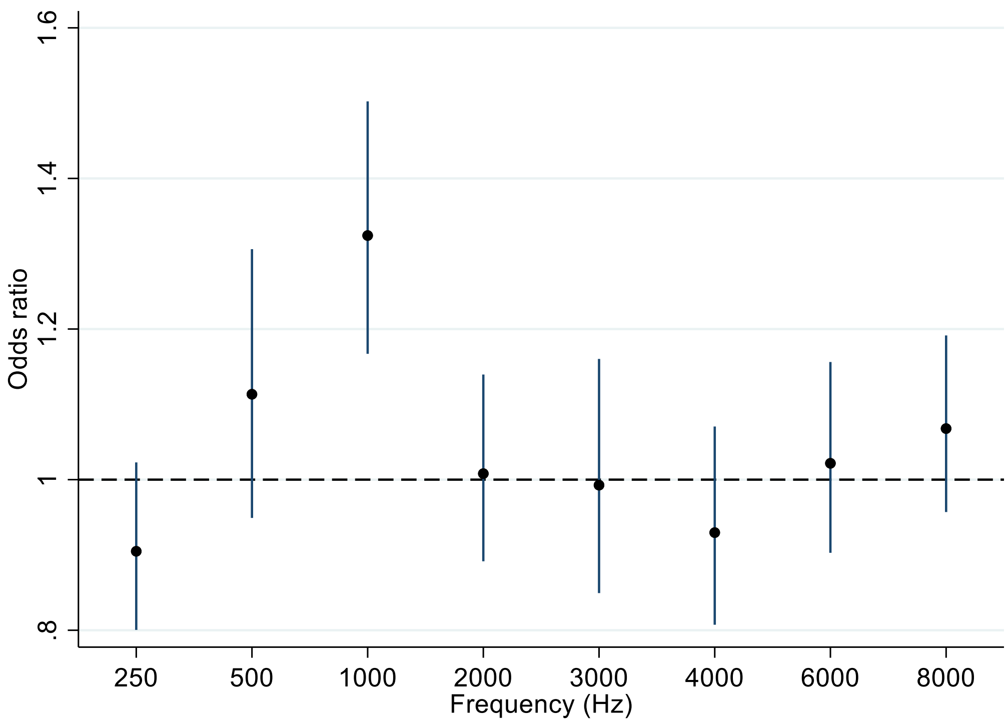

S-Figure 2: Hearing aid benefit estimated by a multivariable ordinal logistic regression model with hearing threshold at the frequencies 250-8 kHz as independent variables. (N = 1,732).). Analyses adjusted for cohort, sex, and age. Odds ratio with 95% confidence intervals.
